# Neuropsychiatric Effects of HIV and Toxoplasma Gondii Encephalitis: A Case Study

**DOI:** 10.1192/j.eurpsy.2025.1689

**Published:** 2025-08-26

**Authors:** T. Incekurk Kizilca, B. Yildirim Cinek

**Affiliations:** 1 Psychiatry, Health Sciences University Erenkoy Mental Health and Neurological Diseases Training and Research Hospital, Istanbul, Türkiye

## Abstract

**Introduction:**

HIV infection can lead to neurological and psychiatric disorders by affecting the central nervous system. Toxoplasma gondii can cause encephalitis in immunocompromised individuals, particularly damaging brain regions such as the frontal cortex and basal ganglia. These neuroanatomical disruptions can influence dopaminergic and serotonergic neurotransmitter pathways, resulting in severe neuropsychiatric symptoms like behavioral disinhibition, personality changes, and psychotic manifestations (Vidal JE. J Int Assoc Provid AIDS Care 2019; 18:2325958219867315).

**Objectives:**

This case seeks to demonstrate the neuropsychiatric effects of HIV and Toxoplasma gondii infections on neuronal function and behavior, emphasizing the importance of continuous assessment and integrated treatment approaches.

**Methods:**

A 49-year-old male patient, with no known medical history, developed Toxoplasma gondii encephalitis after being diagnosed with HIV. Following discharge from the infectious disease ward, the patient exhibited increased irritability, impulsivity, hypersexuality, visual hallucinations, disorganized speech, and risky behaviors, along with cognitive dysfunction and social norm violations. The patient’s symptoms partially improved with a treatment regimen of 15 mg olanzapine and 1000 mg valproic acid. However, the patient’s ongoing course, parkinsonism symptoms emerged, prompting the discontinuation of valproic acid and the consideration of quetiapine. The patient’s follow-up treatment is ongoing.

**Results:**

HIV and Toxoplasma gondii disrupt neuronal function through inflammatory responses and microglial activation in the central nervous system. HIV does not directly damage neurons, but inflammation from cytokines released by microglia can lead to neurodegenerative conditions. Dopaminergic dysfunction is linked to psychotic symptoms, while serotonergic disruptions contribute to depression and anxiety. Frontal lobe and basal ganglia damage impair executive functions (planning, decision-making, impulse control), causing increased impulsivity, risky behaviors, emotional dysregulation, irritability, lack of empathy, behavioral disinhibition, hypersexuality, and cognitive dysfunction. These changes are tied to structural damage in the frontal cortex, necessitating long-term follow-up and comprehensive treatment. Diagnostic imaging often shows white matter lesions, basal ganglia damage, and frontal cortex atrophy. Treatment involves antiretroviral therapy, low-dose antipsychotics, mood stabilizers for managing symptoms (Bartolomé Del Pino LE *et al*. Actas Esp Psiquiatr 2024; 52(2):149-60).

**Conclusions:**

HIV and Toxoplasma gondii infections induce significant central nervous system impairment, leading to neuropsychiatric disorders. This case illustrates their impact on neurotransmitter systems, manifesting as psychosis, cognitive dysfunction, and behavioral disturbances. Continued monitoring and comprehensive treatment are essential.

**Disclosure of Interest:**

None Declared

